# Double Whammy: Why the Underrepresentation of Women among Workplace and Political Decision Makers Matters in Pandemic Times

**DOI:** 10.1017/S1743923X20000628

**Published:** 2020-08-18

**Authors:** Deborah Jordan Brooks, Lydia Saad

**Affiliations:** 1 Dartmouth College; 2 Gallup

**Keywords:** Representation, descriptive representation, female representation, gender, women, female, leader, female leader, decision maker, workplace, work, employment, reopening, policy, Gallup, survey, policy maker, public opinion, COVID-19, coronavirus, pandemic, pandemic politics

## Abstract

In this article, we explore whether women's underrepresentation among political and workplace decision makers may subject female citizens and employees to COVID-19-related decisions that are at odds with their preferences. We find that women overall, as well as female political party members, workers, and workplace leaders in particular, share a distinctively female perspective that more heavily emphasizes caution with respect to COVID-19 compared with men. Given the limited representation of women leaders across most industries and in politics, COVID-19 regulations are thus likely to be less cautious than would be the case if there were an equitable representation of women across leadership roles. We argue that female employees, in particular, face a representational “double whammy” for COVID-19: gender imbalances in workplace leadership create inequities that are compounded—rather than redressed—by unequal political representation. We conclude by addressing how this dynamic may enhance the movement of women away from Republican candidates moving forward.

In recent months, COVID-19 has upended normal daily life, education, and work across the globe. Decisions about school and workplace closures and reopenings have substantial implications for nearly everyone, often pitting health concerns against consumer preferences and economic considerations. In this article, we explore whether the lack of representation of women among both political and workplace decision makers may cause the COVID-19-related interests of female citizens and female workers to be discounted.

We find that women tend to share distinctive perspectives regarding COVID-19 that emphasize caution far more than is the case for their male counterparts, and we show that the gender difference tracks across political parties. We also demonstrate that both female workers and female workplace leaders have fundamentally different preferences than men concerning COVID-19 in the workplace.

In light of the poor representation of women among decision makers in most U.S. industries and in politics, the results of this analysis suggest that regulations about COVID-19-related issues are likely to be less focused on the protection of individuals from the coronavirus than might otherwise be the case if women were equally represented in leadership roles in the United States. Ultimately, we argue that COVID-19 creates a form of interaction of the political and business realms that may be especially harmful to women. Specifically, we conclude that women face a representational “double whammy” regarding COVID-19: their lack of representation among workplace leaders creates problems that are being compounded—rather than redressed—by unequal political representation. We conclude the article by discussing suggestive signs that women have recently been moving away from President Donald Trump at disproportionately higher rates, potentially because of his positioning on COVID-19.

## METHODOLOGY

We examine these issues using large random national samples of Americans collected by Gallup via web surveys. The Gallup Panel is a nationally representative, probability-based survey panel of adults, and the data are weighted according to demographic characteristics to reduce nonresponse bias and improve the representation of the U.S. population.^1^ Studies indicate that probability-based panel polls can provide estimates of public opinion that tend to be at least as accurate as those of traditional telephone polls (MacInnis et al. 2018; Yeager et al. 2011). We use these data for all the analyses that follow, with the analyses of workers and workplace leaders making up a subset of the total sample.

We included data collected from May 18 to June 14, 2020, a time when initial reopening decisions were actively being implemented in many parts of the country. The questions asked in the survey were largely focused on COVID-19 concerns, individual preferences, and workplace structure; there were no questions specifically about COVID-19-related policy on the survey. Especially as an emergent policy area, it seems unlikely that there would be a substantial discrepancy between personal preferences and policy preferences that would further diverge by gender, but this is a potential caveat to note.

## COVID-19 VIEWS BY SEX AMONG U.S. ADULTS

The divergence between women and men across a wide range of questions concerning COVID-19 is striking. Table 1 shows that women express substantially greater levels of concern than men on a wide variety of COVID-19 dimensions.

**Table 1.** COVID-19 views and sex (U.S. adults)

U.S. Adults		MALE	FEMALE	GAP (F-M), % points	Sig	ChiSq

**QUESTION #1:** Which of these do you think is better advice right now for people who do not have symptoms of coronavirus and are otherwise healthy?						
N = 15,145	To stay home as much as possible to avoid contracting or spreading the coronavirus	61%	72%	***12%***	*	0.000
	To lead their normal lives as much as possible and avoid interruptions to work and business	40%	28%	***−12%***	*	
**QUESTION #2:** If there were no government restrictions and people were able to decide for themselves about being out in public, how soon would you return to your normal day-to-day activities?						
N = 15,096	Right now	37%	24%	***−13%***	*	0.000
	After the number of new cases in your state declines significantly	30%	29%	***−1%***		
	After there are no new cases in your state for a period of time	22%	33%	***11%***	*	
	After a coronavirus vaccine is developed	11%	14%	***3%***	*	
**QUESTION #3:** How worried are you that you will get the coronavirus (COVID-19)?						
N = 15,188	Not worried at all	21%	12%	***−9%***	*	0.000
	Not too worried	36%	34%	***−3%***	*	
	Somewhat worried	35%	44%	***9%***	*	
	Very worried	7%	10%	***3%***	*	

* indicates differences between women and men that are significant at .05 or better.

These patterns are notable given that women seem to be carrying a disproportionately greater share of the COVID-19-related care-giving load at home, such as child care and remote schooling (see, e.g., Cohen and Hsu 2020; Miller 2020).

Of course, it is also the case that women tend to be Democrats, and Democrats tend to have more cautious views on COVID-19 relative to Republicans. As such, we examine whether the gender differences shown are a reflection of the partisan gender gap.

Table 2 shows that is not the case. Across both parties and among political independents men tend to be substantially less concerned and cautious about COVID-19 compared with their female copartisans.

**Table 2.** COVID-19 views by sex and party identification (U.S. adults)

		DEMOCRATS					INDEPENDENTS					REPUBLICANS				
U.S. Adults		MALE	FEMALE	GAP (F-M), % points	Sig	ChiSq	MALE	FEMALE	GAP (F-M), % points	Sig	ChiSq	MALE	FEMALE	GAP (F-M), % points	Sig	ChiSq

**QUESTION #1:** Which of these do you think is better advice right now for people who do not have symptoms of coronavirus and are otherwise healthy?																
N = 15,145	To stay home as much as possible to avoid contracting or spreading the coronavirus	94%	95%	***1%***		0.057	59%	70%	***11%***	*	0.000	28%	35%	***8%***	*	0.000
	To lead their normal lives as much as possible and avoid interruptions to work and business	6%	5%	***−1%***			41%	30%	***−11%***	*		73%	65%	***−8%***	*	
**QUESTION #2:** If there were no government restrictions and people were able to decide for themselves about being out in public, how soon would you return to your normal day-to-day activities?																
N = 15,096	Right now	7%	4%	***−2%***	*	0.000	36%	28%	***−8%***	*	0.000	69%	55%	***−14%***	*	0.000
	After the number of new cases in your state declines significantly	36%	32%	***−5%***	*		32%	26%	***−6%***	*		20%	27%	***7%***	*	
	After there are no new cases in your state for a period of time	37%	44%	***7%***	*		21%	34%	***13%***	*		9%	14%	***5%***	*	
	After a coronavirus vaccine is developed	20%	21%	***0%***			11%	12%	***1%***			2%	4%	***2%***	*	
**QUESTION #3:** How worried are you that you will get the coronavirus (COVID-19)?																
N = 15,188	Not worried at all	4.4%	3.5%	***−1%***	*	0.000	23%	14%	***−9%***	*	0.000	37%	25%	***−12%***	*	0.000
	Not too worried	29%	27%	***−2%***			35%	30%	***−5%***	*		46%	47%	***1%***		
	Somewhat worried	55%	54%	***−2%***			35%	47%	***13%***	*		15%	24%	***10%***	*	
	Very worried	11%	15%	***4%***	*		8%	8%	***0%***			2%	3%	***1%***	*	

* indicates differences between women and men that are significant at .05 or bett

Appendix A in the supplementary material online shows a corresponding pattern of results based on multivariate analyses with the inclusion of a series of control variables.

Our results match up with research on previous respiratory diseases across the globe. Specifically, Moran and Del Valle (2016) conducted a meta-analysis of 85 studies of responses to respiratory outbreaks around the world and found that women were approximately 50% more likely to practice nonpharmaceutical preventative behaviors (i.e., using masks, extra handwashing, etc.) during outbreaks than men.

Establishing why sex differences exist in responses to epidemics is beyond the scope of this article, although our findings suggest some possibilities. Studies have found that the views of men and women tend to differ when risk is involved. In particular, scholars have found that women have a greater preference for risk reduction on environmental issues when safety risks are involved (Bord and O'Connor 1997; Norrander 2008). Our results seem consistent with a lower perception of risk by men than by women, since we find that women are not just more likely to prefer a cautious approach to COVID-19 but also more concerned than men about catching the coronavirus, even though men are far more likely die from it (Reeves and Ford 2020).

## WORKPLACE DIFFERENCES IN COVID-19 PREFERENCES BETWEEN MEN AND WOMEN

Given the rapid spread of COVID-19, workplace decisions have critical importance for the economic livelihood and safety of employees. As a result, we examine whether there is a gender gap among workers regarding responses to COVID-19 and, in turn, whether differences exist between workplace leaders versus nonleader employees.

Questions regarding COVID-19 in the workplace were asked of a subset of the Gallup panelists represented in the analyses who had been employed over the seven days prior to the day the survey was taken. When asked which option describes their role or job responsibilities at work, 21% of employed male respondents and 13% of employed female respondents selected “I am a leader who manages others” (coded as “leaders”), while the balance selected nonleadership descriptions of their role at work (coded as “workers”).^2^

The “Workers” column of Table 3 shows a persistent gender gap. Female workers are significantly more concerned about the safety of returning to work, are more likely to prefer to continue working at home because of COVID-19-related concerns, and anticipate a longer duration of continued disruption.

**Table 3.** COVID-19 views by sex for workers versus workplace leaders (employed U.S. adults)

		Workers					Workplace Leaders				
Employed U.S. Adults		MALE	FEMALE	GAP (F - M)	Sig	ChiSq	MALE	FEMALE	GAP (F - M)	Sig	ChiSq

**QUESTION 1:** How concerned are you about being exposed to coronavirus at your place of work?											
N = 7,289 (Male Leaders = 914, Female Leaders = 395)	Very concerned	12%	19%	**8%**	*	0.000	7%	11%	**5%**	*	0.000
	Moderately concerned	28%	35%	**7%**	*		32%	39%	**7%**	*	
	Not too concerned	33%	26%	**−7%**	*		35%	31%	**−4%**		
	Not concerned at all	28%	20%	**−7%**	*		27%	19%	**−8%**	*	
**QUESTION #2**: I can now return to work safely											
N = 6,571 (Male Leaders = 847, Female Leaders = 380)	1 - Strongly Disagree (i.e. very unsafe)	13%	30%	**17%**	*	0.000	11%	23%	**11%**	*	0.000
	2	16%	18%	**3%**	*		10%	10%	**0%**		
	3	19%	20%	**1%**			18%	25%	**7%**	*	
	4	18%	15%	**−3%**	*		18%	16%	**−2%**		
	5 - Strongly Agree (i.e. very safe)	34%	17%	**−17%**	*		43%	26%	**−17%**	*	
**QUESTION #3**: How long do you think the level of disruption occurring to travel, school, work and public events in the U.S. will continue before it starts to improve?											
N = 7291 (Male Leaders = 915, Female Leaders = 396)	A few more weeks	20%	12%	**−8%**	*	0.000	22%	10%	**−12%**	*	0.000
	A few more months	31%	26%	**−5%**	*		28%	29%	**1%**		
	For the rest of this year	30%	38%	**8%**	*		34%	30%	**−5%**		
	Longer than this year	20%	24%	**5%**	*		16%	31%	**15%**	*	
**QUESTION #4:** Once restrictions on businesses and school closures are lifted, if your employer left it up to you, would you prefer to (return to working at office vs. work remotely; follow up on “work remotely” asks preference vs. Covid)											
N = 3,447 (Male Leaders = 458, Female Leaders = 229)	Return to working at your office or workplace as much as previously did	38%	26%	**−12%**	*	0.000	53%	43%	**−11%**	*	0.033
	Work remotely as much as possible as preference	39%	34%	**−5%**	*		29%	32%	**3%**		
	Work remotely as much as possible due to Covid	23%	39%	**17%**	*		18%	26%	**8%**	*	

* *indicates gender differences that are significant at .05 or better*.

A related question is whether the preferences of female workers would be better represented by female workplace leaders as decision makers, which requires first identifying whether a similar gender gap regarding caution toward COVID-19 is also observed among their leaders.

Table 3 confirms that female workplace leaders are also more likely than their male counterparts to take a cautious approach to COVID-19.

The multivariate analyses in Appendix B allow us to confirm that gender is a significant determinant of COVID-19 views in the workplace both with and without interactions between sex and leadership. Controls were included for partisanship (as dummies for “Republican” and “Independent”), low-density population (rural/small town), married, parent, college graduate, race/ethnicity (dummy coded as white, non-Hispanic), age 65 or older, high health risk for COVID-19, high income (household income of $120,000 or more) and low income (household income of $24,000 or less), essential worker,^3^ and week of survey administration.

In short, all of the analyses point in the same direction: male workers are far more bullish about returning to work life as usual than their female colleagues, and similarly gendered patterns exist among workplace leaders.

## POTENTIAL WORKPLACE IMPLICATIONS

From the foregoing analyses, we can conclude that female business leaders would be likely to voice different perspectives on COVID-19-related workplace policies than their male colleagues. But how likely are they to have an effective platform for doing so?

Just 30% of the self-described workplace leaders in our sample were female, reflecting a strong male skew in leadership across the majority of U.S. industries and workplaces; moreover, gender imbalances in leadership tend to be even greater at the highest levels of decision-making for the largest employers. For example, just 7.4% of the CEOs of Fortune 500 companies are female (Hinchliffe 2020; see also Yanosek, Ahmad, and Abramson 2019). It is notable as well that even in fields that are mostly composed of female workers, the leadership still skews heavily male: for example, only 3% of health care CEOs and medical division chiefs are female (compared with 80% of health care workers), and only one-quarter of school district superintendents are female, while just over three-quarters of K–12 teachers are women (Rotenstein 2018; Superville 2016).

Those mostly male workplace leaders are probably unlikely to feel strong pressure to factor in the distinctive COVID-19-related preferences of female workers. Leaders outside of politics do not tend to be democratically elected, generally holding powers more akin to autocrats or even dictators (Wadhwa 2016). Ultimately, the exclusion of women from leadership across so many industries strongly suggests that female employees are likely to find themselves pressured or even required to return to workplaces sooner than it feels safe to them, and, by extension, once returning, they may also find fewer safety precautions in place than they prefer.

## POTENTIAL POLITICAL IMPLICATIONS

The political realm could potentially serve as a bulwark to prevent female workers from working under conditions they deem unsafe. Unfortunately, women are also descriptively underrepresented across all levels of politics; moreover, the vast majority of female office holders hail from the Democratic Party (for the most recent numbers, see CAWP 2020). There is active debate about the degree to which co-partisanship versus shared gender most effectively promotes the interests of women. But to the extent that descriptive representation does matter, there is reason to think that healthcare might be one of the policy arenas where it might matter a good deal (Swers 2016).

Might this pandemic cause women—and perhaps Republican-leaning working women in particular—to become especially concerned about the relative lack of female decision makers both at work and in politics? If Republican leaders continue to minimize the need for safety measures around COVID-19 (Hellmann 2020; Wildermuth 2020), could it lead to a further shift among women in the short term toward a vote for Joe Biden over Trump, or away from the Republican party in the long term?

Partisanship tends to be “sticky,” and candidate preference tends to be closely tied to partisanship (see, e.g., Green, Palmquist, and Schickler 2002). These are not normal times, however, and COVID-19 is not a normal issue. The degree to which this pandemic creates what for many may feel like fundamental concerns about economic and corporeal survival—not just for individuals but for entire households and communities—should not be underestimated. It is thus interesting that some early indications have emerged that Trump has been losing support among women to an even greater extent in recent months. For example, some polls found that the drop in those preferring Trump over Biden was particularly marked between March/April and May/June 2020 (Cohn 2020). With other factors in play during that same time period, a clear causal relationship cannot be isolated; moreover, those results do not isolate female employees specifically. However, results such as those provide some suggestive evidence that the Republican Party may be facing a new cleavage that may further peel away female support for Republican candidates, thereby potentially expanding an already considerable partisan gender gap.

## Footnotes

1. More information about the Gallup Panel can be found at https://www.gallup.com/174158/gallup-panel-methodology.aspx (accessed September 2, 2020).

2. The specific options presented to respondents were: 1. “I am a leader who manages others”; 2. “I am a manager/supervisor whose main responsibilities are the work output of other people”; 3. I am a project manager who is responsible for other people's work; 4. “I am an individual contributor responsible for my own work or output.” In our analyses, 1 = ”leader” and 2, 3, and 4 = “worker.”

3. Men and women, on average, tend to hold different types of roles in different industries; that could be a confound if women are more likely to be represented in the ranks of frontline workers. By controlling for “essential workers” based on self-report (i.e., “Is your job something your state government considers an essential job function that is exempt from stay-at-home orders?”), we are able to show that it does not negate the gender gap. Moreover, its presence in the equation does not significantly affect any of the core findings.

## Supplementary material

For supplementary material accompanying this paper visit https://doi.org/10.1017/S1743923X20000628. click here to view supplementary material
