# Supplementary material for: Double Whammy: Why the Underrepresentation of Women among Workplace and Political Decision Makers Matters in Pandemic Times
Source: Politics & Gender. 2020 Aug 18:1–13. doi: 10.1017/S1743923X20000628 (PMC7562855; doi:10.1017/S1743923X20000628)
Supplement: Supplementary file 1 [file S1743923X20000628sup001.pdf]

Appendix A

Covid-19 and U.S. Public Opinion (Multivariate Analyses)

We conducted multivariate analyses to confirm that what appears to be a strong relationship in the crosstabular results holds up with controls, and to examine the interaction between sex and partisanship.

Control variables for all of the models consist of partisanship (as dummies for “Republican” and “Independent”), low density population (rural/small town), married, parent, college grad, race/ethnicity (dummy coded as white, non-Hispanic), age 65+, high health risk for Covid, employed (in the last seven days), high income (household income of $120,000 or more) and low income (household income of $24,000 or less), and week of survey administration (to control for possible noise associated with changes over the four weeks of survey administration).

All dependent variables in the multivariate analysis in Table A3 are coded such that positive numbers denote a lack of concern about Covid (i.e., pushing forward) while negative numbers indicate concern (i.e., pulling back). Because each of the dependent variables varies in terms of its construction, analytical techniques appropriate for each type of D.V. are utilized for each analysis.

Model #1 shows that gender is a significant determinant of Covid views across all of the measures, confirming that the gender differences are not functioning as a proxy for the partisan gender gap.

Model #2 examines the interaction between sex and party. Two of the three analyses produce results that suggest that the gender differences do not vary significantly for women vs. men by party. The sole exception is Question #1. The sex coefficient remains directionally consistent, but the standard error increases, resulting in a lack of statistical significance once the interaction terms are added. This is likely an artifact of the near-unanimity among Democrats on that particular question, given that, at the time of the survey, 94% of Democratic males and 95% of Democratic females felt that staying home as much as possible was the best advice for healthy people.

U.S. Adults		Q#1 - Best Advice		Q#2 - If no restrictions		Q#3 - How Worried	
		Logit		OLS		OLS	
		Coef.	*SE*	*Coef.*	*SE*	*Coef.*	*SE*
Model #1	Female	-0.29 *	*(.102)*	-0.14 *	*(.032)*	-0.15 *	*(.029)*
	Republican	3.46 *	*(.150)*	1.14 *	*(.036)*	0.79 *	*(.034)*
	Independent	2.09 *	*(.149)*	0.50 *	*(.043)*	0.35 *	*(.036)*
Model #2	Female	-0.25	*(.253)*	-0.09 *	*(.046)*	-0.08 *	*(.040)*
	Republican	3.47 *	*(.211)*	1.18 *	*(.050)*	0.85 *	*(.047)*
	Independent	2.13 *	*(.208)*	0.54 *	*(.058)*	0.40 *	*(.050)*
	Republican X Female	-0.01	*(.293)*	-0.07	*(.068)*	-0.11	*(.064)*
	Independent X Female	-0.09	*(.297)*	-0.09	*(.085)*	-0.11	*(.073)*
	n=	*12,121*		*12,089*		*12,147*	

** = Coefficient is significant at .05. All variables are coded such that negative coefficients indicate greater concern about Covid. See list of control variables in text.*

Appendix B

Covid-19 and Worker vs. Workplace Leader Opinion (Multivariate Analyses)

Just as with the U.S. adult analysis in Appendix A, we conducted multivariate analyses to confirm that what appears to be a strong relationship in the crosstabular results for workers holds up with controls, and to see if gender differences were comparable across workers and leaders.

The only difference from the controls used for U.S. adults was the exclusion of “employed” (since that was the condition required for being included in this sub-sample), and the addition of the control for essential workers.[i] Otherwise, the control variables are the same as in Appendix A. They include partisanship (as dummies for “Republican” and “Independent”), low density population (rural/small town), married, parent, college grad, race/ethnicity (dummy coded as white, non-Hispanic), age 65+, high health risk for Covid, high income (household income of $120,000 or more) and low income (household income of $24,000 or less), and week of survey administration (to control for possible noise associated with changes over the four weeks of survey administration).

All dependent variables in the multivariate analysis in these tables are coded such that positive numbers denote lack of concern about Covid (i.e., pushing forward) while negative numbers indicate greater concern (i.e., pulling back). Because each of the dependent variables varies in terms of its construction, analytical techniques appropriate for each type of D.V. are utilized for each analysis.

Employed U.S. Adults		**Q#1 - Work concern**		**Q#2 - Return Safely**		**Q#2 - How Long**		**Q#4 - Office vs Home**	
		OLS		OLS		Ordered Logit		Logit ^	
		Coef.	*SE*	*Coef.*	*SE*	*Coef.*	*SE*	*Coef.*	*SE*
Model #1	Female	-0.23 *	*(.048)*	-0.30 *	*(.065)*	-0.24 *	*(.097)*	-0.53 *	*(.174)*
	Leader	0.01	*(.060)*	0.19 *	*(.084)*	-0.14	*(.128)*	0.22	*(.267)*
Model #2	Female	-0.24 *	*(.052)*	-0.34 *	*(.069)*	-0.22 *	*(.104)*	-0.59 *	*(.186)*
	Leader	-0.05	*(.075)*	0.09	*(.104)*	-0.08	*(.165)*	-0.01	*(.370)*
	Female X Leader	0.13	*(.121)*	0.28	*(.171)*	-0.17	*(.258)*	0.46	*(.516)*

** = Coefficient is significant at .05. All variables are coded such that negative coefficients indicate greater concern about Covid. See list of included control variables in text.*

^ Because the matter of primary interest for Q#4 is whether a respondent prefers to work from home due to Covid, work from home due to non-Covid preference and work from office/workplace were collapsed into the "0" category.

Summary of Findings

See the text for more discussion of these results. The bottom line is that, with an array of controls, the patterns implied by the crosstabular results hold. Men are significantly less likely to be concerned about contracting Covid at work, they are much less likely to expect a protracted disruption from Covid, and they are much less likely to state a preference for working from home due to Covid-related reasons. Additionally, the lack of significance for the interactions suggests that degree of difference between male and female leaders is not significantly different than that of non-leader workers.

All of the evidence points towards the finding that men have much less concern about Covid than women across the general population, across parties, as workers, and as workplace leaders.

---

[i] Coding for "essential worker" is based on a question asking respondents, "Is your job something your state government considers an essential job function that is exempt from stay-at-home orders?" Findings for the core variables of interest are the same regardless of whether this variable is included or excluded.
